# Empagliflozin-Associated Pancreatitis in the Setting of Hyperglycemic Hyperosmolar Syndrome

**DOI:** 10.7759/cureus.60935

**Published:** 2024-05-23

**Authors:** Jenna C Borrelli, Martine A Cioffi, Nora Martini, Mark Samarneh

**Affiliations:** 1 Internal Medicine, Lake Erie College of Osteopathic Medicine, Yonkers, USA; 2 Internal Medicine, St. John's Riverside Hospital, Yonkers, USA; 3 Internal Medicine/Nephrology, Riverside Health System, Yonkers, USA

**Keywords:** drug-induced pancreatitis, sodium-glucose cotransporter-2 (sglt2) inhibitors, hyperosmolar hypoglycemic syndrome, empagliflozin, pancreatitis causes

## Abstract

Acute pancreatitis is a prevalent gastrointestinal condition in the United States, with approximately 130,000 new cases annually, displaying a rising incidence. Severe cases, constituting 20% of instances, necessitate intensive care unit admission, associated with elevated mortality rates. While gallstones and chronic alcohol use are primary causes, certain medications, including ACE inhibitors, statins, hormone-replacement therapies, diuretics, hypoglycemic agents, and steroids, can induce pancreatitis. Notably, recent reports link empagliflozin, an SGLT-2 inhibitor used in managing type 2 diabetes, to pancreatitis, a rare complication in this drug class. This article details a case study of a 57-year-old African American man presenting with hyperglycemic hyperosmolar syndrome due to empagliflozin-induced pancreatitis, a novel sequela. The discussion underscores the role of sodium-glucose cotransporter-2 (SGLT-2) inhibitors in diabetes management, emphasizing their advantages and associated complications. This report adds a unique dimension to the literature, emphasizing the importance of prompt identification and cessation of culpable agents to prevent adverse outcomes.

This article aims to comprehensively address the prevalence and increasing incidence of acute pancreatitis in the United States. This report aims to assist healthcare professionals in recognizing and discontinuing causative agents, thereby providing valuable insights into the comprehension of drug-induced pancreatitis.

## Introduction

Acute pancreatitis is the leading cause of gastrointestinal hospital admissions in the United States [1]. Presentations could range from mild, such as edematous pancreas, to severe, such as necrotizing pancreatitis, and may even involve multi-organ disease and require intensive care unit admission. In the United States, there are approximately 130,000 new cases of acute pancreatitis annually and the incidence is increasing over time likely due to obesity and predisposition to gallstones [2]. Although only 20% of cases progress to an ICU admission, these severe cases are associated with increased hospital length of stay, procedures, and management, with an increase in mortality up to 30% [3]. 

There are many known causes of acute pancreatitis, the most common being gallstones and chronic alcohol use disorder. Several medications also have pancreatitis listed as a documented side effect. These medications include angiotensin-converting enzyme (ACE) inhibitors, statins, hormone-replacement therapies, diuretics, hypoglycemic agents, and steroids [4]. Concerning the hypoglycemic agents, glucagon-like peptide-1 (GLP-1) mimetics have primarily caused exacerbations of acute pancreatitis [4]. However, recent case reports have identified another possible medication class associated with pancreatitis, that being the sodium-glucose cotransporter-2 (SGLT-2) inhibitors [5]. The presumed mechanism of drug-induced pancreatitis is through the cytotoxic effects of either the drug itself or its metabolites [6]. This case not only recognizes the recently identified link between SGLT-2 inhibitors and pancreatitis, but it also depicts the accelerated sequela into hyperglycemic hyperosmolar syndrome (HHS) caused by pancreatitis. HHS encompasses uncontrolled blood glucose levels, commonly seen in type 2 diabetics. This syndrome typically presents with increased urination (polyuria), increased water intake (polydipsia), lethargy, and possibly neurologic changes. As seen in this case, pancreatitis can lead to HHS by damaging the exocrine pancreatic function and decreasing insulin levels [7]. If left untreated, HHS can rapidly progress from lethargy to coma, due to the severe dehydration and hyperglycemic effects. This study discusses the effects of drug-induced pancreatitis on plasma glucose levels and its subsequent complications. 

## Case presentation

A 57-year-old African American man presented with worsening epigastric pain, intermittent nausea, and several episodes of non-bloody, non-bilious emesis for the past five days. His past medical history is significant for decompensated heart failure (ejection fraction of 30%) requiring a temporary left ventricular assist device, cardiac arrest, newly diagnosed type 2 diabetes mellitus, and hypertension. In the emergency department, the patient was normotensive and normothermic, with a normal pulse rate, regular respirations, and saturating appropriately. Physical exam was notable for tenderness in the epigastric region and mild abdominal distention.

**Figure 1:**
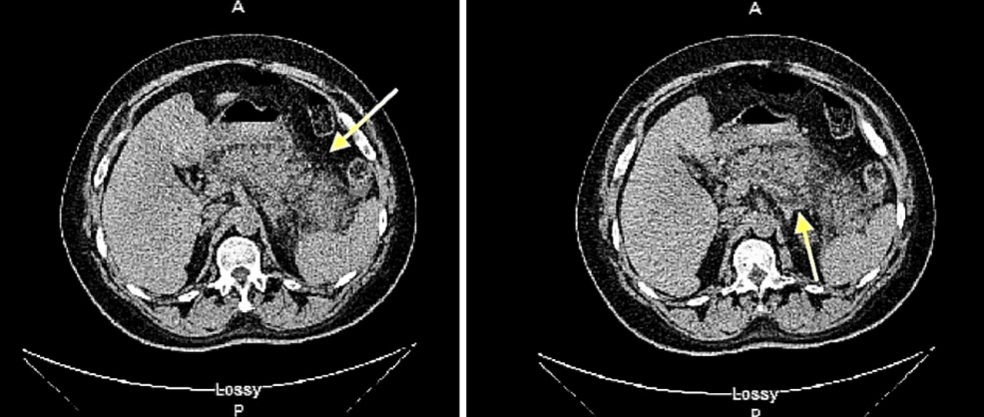
Abdominal CT scan Abdominal CT depicting an enlarged pancreas, significant peripancreatic edema/stranding (yellow arrow), and peripancreatic fluid extending to the left pararenal space and paracolic gutter, all of which are classic signs of pancreatitis.

On admission, labs were notable for plasma glucose of 1261 mg/dL, beta-hydroxybutyrate of >30 mg/dL, lactic acid of 4.6 mmol/L, anion gap of 23 mmol/L, serum lipase of 1006 U/L, and triglyceride level mildly elevated at 406 mg/dL. Urinalysis demonstrated 3+ glucose and trace ketones. The markedly elevated glucose level, high anion gap, and mild ketosis in a type 2 diabetic led to a diagnosis of HHS (Table 1). The significantly elevated lipase level and epigastric pain alone are sufficient to diagnose acute pancreatitis, which was further confirmed by imaging (Figure 1).

**Table 1:** Displayed lab values from admission to discharge, with significant peaks *Beta-hydroxybutyrate, urinalysis, and hemoglobin A1c% only measured on admission. **Last recorded lactic acid value prior to discharge, not on the day of discharge. Reference values: Plasma glucose: 125 mg/dL or less, beta-hydroxybutyrate: <9 mg/dL, lactic acid: <2 mmol/L, anion gap: 4-12 mmol/L, serum lipase: 0-160 U/L, triglycerides: <150 mg/dL, AST: 8-48 U/L, ALT: 7-55 U/L, hemoglobin A1c: <5.7%

Labs	Admission	Peak	Discharge
Plasma glucose	1261 mg/dL	1261 mg/dL	196 mg/dL
Beta-hydroxybutyrate*	33.5 mg/dL	-	-
Lactic acid	4.6 mmol/L	4.6 mmol/L	2.3 mmol/L**
Anion gap	23 mmol/L	23 mmol/L	9 mmol/L
Serum lipase	1006 U/L	7534 U/L	917 U/L
Triglycerides	406 mg/dL	406 mg/dL	234 mg/dL
AST	13 U/L	27 U/L	19 U/L
ALT	28 U/L	28 U/L	18 U/L
Urinalysis*	3+ glucose, trace ketones	-	-
Hemoglobin A1c%*	12.3%	-	-

A detailed history revealed that the patient's type 2 diabetes had been managed exclusively with empagliflozin for the past year, with confirmed medication compliance. Other major causes of acute pancreatitis, including alcohol use, cholelithiasis, hypertriglyceridemia, hypercalcemia, steroid use, and other medications, were systematically ruled out. The patient denied any history of alcohol use disorder, recent alcohol intake, steroid use, or recent prescriptions associated with pancreatitis. The patient's serum calcium level was within normal limits. Although the patient exhibited elevated serum triglyceride levels, they were not sufficiently high to induce acute pancreatitis (Table 1). Abdominal ultrasound showed a normal gallbladder, no cholelithiasis, no gallbladder wall thickening, and a negative sonographic Murphy’s sign. Abdominal CT demonstrated an enlarged pancreas, significant peripancreatic edema/stranding, and peripancreatic fluid extending to the left pararenal space and paracolic gutter (Figure 1).

In the emergency department, the patient was started on a weight-based insulin regimen, lactated ringers for rehydration, and was admitted to the intensive care unit for management of acute pancreatitis with HHS. The patient’s symptoms improved within 11 days and his laboratory values normalized. The patient was discharged home on an insulin regimen, and empagliflozin was discontinued. 

## Discussion

The SGLT-2 inhibitors, canagliflozin, dapagliflozin, and empagliflozin, are FDA-approved oral hypoglycemic agents. The mechanism of action of these medications works by inhibiting the SGLT-2 in the proximal tubule, causing glucosuria and leading to lower blood glucose levels [8]. The SGLT-2 expressed in the proximal tubule mediates the reabsorption of 90% of the filtered glucose. By inhibiting this cotransporter, the drug reduces blood glucose by increasing urinary glucose excretion. The American Diabetes Association recommends that this medication be used after healthy lifestyle changes and diet modification, and medical intervention with metformin was proven insufficient in controlling the patient’s hyperglycemia [8]. There are numerous advantages of using an SGLT-2 inhibitor compared to other diabetes medications, most of which stem from its mechanism of being independent of insulin levels and sensitivity. By bypassing insulin, patients are at lower risk of hypoglycemia, weight gain, and hepatic injury compared to other diabetic medications. 

SGLT-2 inhibitors play a crucial role for older adults with type 2 diabetes by enhancing glycosuria, reducing glycemia, and ensuring effective glycemic control. Beyond its primary focus on diabetes, empagliflozin demonstrates positive outcomes in cardiovascular diseases, chronic kidney disease, and heart failure, showing efficacy irrespective of ejection fraction. It further showcases renoprotective effects, reducing the risk of nephropathy and demonstrating effectiveness in managing diabetic nephropathy while lowering the risk of kidney-related complications. Empagliflozin also exhibits cardioprotective effects, lowering the risk of major adverse cardiovascular events and hospitalizations due to heart failure, thereby offering protection against both cardiovascular and renal disorders [9]. Its metabolic and mitochondrial effects contribute to reducing blood pressure, aiding weight loss, and improving cardiomyocyte Ca2+ handling, with positive effects on mitochondrial architecture. Additionally, empagliflozin is associated with hematologic benefits, including increased hematocrit and hemoglobin levels, potentially slowing the progression of chronic kidney disease [9]. The medication also demonstrates anti-inflammatory effects, suppressing inflammation in patients with diabetes. 

Their well-studied adverse effects include genitourinary infections, diabetic ketoacidosis, and acute kidney injury. Though pancreatitis is common among other diabetes medications, such as GLP-1 mimetics and dipeptidyl peptidase-4 (DPP-4) inhibitors, it is not recognized by the FDA as an adverse effect of SGLT-2 inhibitor use. Over the last decade, there have been rare reported cases of pancreatitis linked to canagliflozin, dapagliflozin, and empagliflozin [5]. However, this case is interesting because the empagliflozin-induced pancreatitis led to the complication of HHS. The pathophysiology behind this is that acute pancreatitis leads to stress hyperglycemia. This phenomenon occurs due to the overactivation of the sympathetic nervous system, causing elevated glucagon [7]. The extensive edema and ischemia also cause decreased insulin production. In a patient without diabetes, the body may either compensate properly or potentially lead to a diabetic state. However, in a patient with preexisting diabetes, pancreatitis may accelerate severe complications. 

This report depicts another case of empagliflozin-associated pancreatitis, except with a sequela of HHS, which has not been previously documented. It is imperative that healthcare professionals possess the ability to promptly discern any culpable agents, allowing for their cessation, and ultimately allowing prevention of any further detrimental effects. 

## Conclusions

Acute pancreatitis often leads to systemic inflammatory disease, potentially complicated by multi-organ system involvement if left untreated. While this disease typically has a classic presentation, there are various etiologies contributing to the onset of the disease. It is vital that medical providers identify offending agents as early as possible for prompt discontinuation to prevent further damage or subsequent episodes. 

This case impacts the future management of diabetes by highlighting the importance of medication reconciliation. If physicians make a priority of reviewing medications and their potential side effects at each visit, complications can be identified earlier or potentially avoided, leading to better healthcare management. Regularly reviewing a patient's medication list should not be overlooked as a routine task; it is a vital step in ensuring patient safety and effective diabetes management. While the FDA is investigating the possible link between acute pancreatitis and SGLT-2 inhibitors, clinicians should be aware of this interrelation to enhance the management of their diabetic patients. This proactive effort may lead to improved patient outcomes.
